# Lamarckian Evolution of Simulated Modular Robots

**DOI:** 10.3389/frobt.2019.00009

**Published:** 2019-02-18

**Authors:** Milan Jelisavcic, Kyrre Glette, Evert Haasdijk, A. E. Eiben

**Affiliations:** ^1^Faculty of Science, Vrije Universiteit Amsterdam, Amsterdam, Netherlands; ^2^RITMO, Department of Informatics, University of Oslo, Oslo, Norway

**Keywords:** evolutionary robotics, artificial life, Lamarckian evolution, modular robots, online learning, embodied evolution

## Abstract

We study evolutionary robot systems where not only the robot brains but also the robot bodies are evolvable. Such systems need to include a learning period right after ‘birth' to acquire a controller that fits the newly created body. In this paper we investigate the possibility of bootstrapping infant robot learning through employing Lamarckian inheritance of parental controllers. In our system controllers are encoded by a combination of a morphology dependent component, a Central Pattern Generator (CPG), and a morphology independent part, a Compositional Pattern Producing Network (CPPN). This makes it possible to transfer the CPPN part of controllers between different morphologies and to create a Lamarckian system. We conduct experiments with simulated modular robots whose fitness is determined by the speed of locomotion, establish the benefits of inheriting optimized parental controllers, shed light on the conditions that influence these benefits, and observe that changing the way controllers are evolved also impacts the evolved morphologies.

## 1. Introduction

Evolutionary methods provide a successful approach to designing robots (Nolfi and Floreano, 2000; Bongard, 2013; Vargas et al., 2014; Doncieux et al., 2015). The central premise behind evolutionary robotics is the proven power of natural evolution to generate viable life forms for a vast variety of possible environments. For robotics, this means that using artificial evolution it is plausible that we can design robots for (m)any possible environment(s) and task(s). Historically, evolutionary robot design has focused on the evolution of robot controllers for fixed, hand-designed morphologies and the evolution of morphologies has not received much attention. Arguably, this attitude is inherently missing out, because robot behavior is determined not only by the controller but also by the morphology.

In this paper, we consider the evolution of morphologies as well as controllers in a system of modular robots. This means that the evolving population is made up of robots and adding a new individual to the population amounts to the creation of a new robot. The ‘birth' of a robot starts off its lifetime period that finishes when the robot is removed from the population. In this period the robot can interact with its environment and possibly reproduce, that is, undergo crossover and/or mutation resulting in a new robot. We assume that the controller of a robot can be adjusted during its lifetime, but its morphology does not change. Then we can make a general distinction between evolution and learning regardless the specific algorithmic details. The evolutionary process searches the set of all robots within the given design space. One step in this search process is the creation of a robot with a new morphology (body) and controller (brain). In contrast, the learning algorithm attempts to improve the controller of a robot during its lifetime. Hence, it operates in the space of controllers that fit the given body.

A fundamental feature of such systems is that the morphology of the offspring can and will be different from that of the parent(s). Consequently, “newborn” robots necessarily need to learn an appropriate controller, because in general it cannot be assumed that an inherited controller matches the inherited morphology. For this reason, we argue that any system of morphologically evolving robots must include a learning phase for “newborn” robots. Therefore, we adopt a system architecture that does contain such a phase. This architecture, called the Triangle of Life, was introduced in Eiben et al. (2013) and used in several experimental studies that addressed the task of gait learning (Rossi and Eiben, 2014; Jelisavcic et al., 2016; Weel et al., 2017). Note that the choice of the gait learning task is not arbitrary. Locomotion is one of the most fundamental robot skills that depends on the morphology, and gait learning is the most elementary form of locomotion without any specific direction to follow or target to approach. As observed by the research community including ourselves, the gait learning process can take a significant amount of time and effort for a robot. This raises the principal research question behind this paper:

Could the learning process be improved by making learned controllers inheritable and bootstrapping infant learning with the learned controllers of the parents?

Technically speaking, we are interested in Lamarckian evolution, where a robot can pass on characteristics acquired during its lifetime to its offspring, not by communication (that would be “teaching”), but through genetic inheritance (Hinton and Nowlan, 1987; Burkhardt, 2013). Although the idea seems straightforward, until recently there has been hardly any research into the effects of Lamarckian set-ups combining morphological evolution and lifetime learning of robot controllers. Jelisavcic et al. (2017c) report preliminary findings that indicate that such a Lamarckian set-up can improve performance. A more in-depth follow-up investigation reported a strong correlation between the advantage of inheriting parental controllers and the amount of retained parental controller structure (Jelisavcic et al., 2017b).

These studies raise new questions and motivate further research into the combination of Darwinian evolution of morphologies^1^ and Lamarckian evolution of controllers. In this paper, we address three specific research questions in the context of modular robots and locomotion as the primary task:

1. Does the inheritability of learned features provide an advantage?
2. What does this advantage depend on?
  For instance, does it depend on:
  - The morphological similarity between parents and offspring?
  - The morphological similarity between parents?
  - The stage of the evolutionary process, e.g., is the effect of inheritable controllers different in early and late generations?
3. Are the morphologies to which robots evolve different for Darwinian and Lamarckian evolution?

## 2. Related Work

Evolutionary Robotics is the field that lies at the intersection between of evolutionary computing and robotics (Nolfi and Floreano, 2000; Wang et al., 2006; Floreano et al., 2008; Trianni, 2008; Doncieux et al., 2011, 2015; Bongard, 2013; Vargas et al., 2014). The field aims to apply evolutionary computation techniques to evolve the design, controllers, or both, for real and simulated autonomous robots.

### 2.1. Simultaneous Evolution of Morphology and Control

A robot's behavior is the result of the interaction between its morphology, controller, and environment (Pfeifer and Iida, 2004). Evolutionary robotics offers a methodology to consider the development and adaptation of robot morphology and control holistically (Eiben and Smith, 2015). Simultaneous evolution of morphology and control was introduced with Sims' simulated virtual creatures (Sims, 1994) and has been investigated without regards to physically producible results many times since then. One notable example is the work of Cheney et al. (2013), using a voxel-based substrate to evolve soft-bodied virtual robots.

Lipson and Pollack (2000) first demonstrated that this approach is also applicable in systems where the final (i.e., after evolution has run its course) results are realized and evaluated as actual physical robots, with substantial research revisiting this approach. For example, generative encodings have been explored and resulted in physically instantiated robots in Hornby et al. (2003) and Samuelsen and Glette (2015), resulting in regular, symmetrical, and insect-like bodies and behaviors. Moreover, robot shapes have been optimized in Clark et al. (2012) and Corucci et al. (2015), resulting in dynamic body-brain behavior for aquatic robots. There have also been some approaches targeting modular robotic systems, such as robotic manipulator design in Chocron and Bidaud (1997), and a multi-purpose approach in Faíña et al. (2013). In Marbach and Ijspeert (2005), modular robots had their morphology and control parameters evolved offline, before being subject to on-line control adaptation using a fast-converging function optimization algorithm. We have also recently seen experiments where even the robot morphologies have evolved in a real-world setup; using an external robotic arm to assemble modules in Brodbeck et al. (2015), or a self-reconfiguring robot in Nygaard et al. (2018).

The simultaneous development of robot morphologies and control systems is a difficult task, and we have only seen relatively simple results so far, as noted by Lipson et al. (2016). As Pfeifer and Iida (2005) conclude, “morphological computation is about connecting body, brain and environment.” Some of the difficulty is due to the increased dimensionality of the search, but a more insidious aspect may be the increased ruggedness of the search space: a small mutation in the morphology can easily offset the performance of the controller-body combination found earlier. Lipson et al. (2016) illustrate this by casting the morphology as a physical interface between controller and environment; the variation operators that generate a new individual can then be seen as “scrambling” this interface. An obvious remedy would be to allow the controller to adjust to the new morphology, on a different timescale from the morphological changes—i.e., to enable lifetime learning for new robot bodies.

### 2.2. Locomotion Controller Approaches

Robotic locomotion requires the creation of rhythmic patterns which satisfy multiple constraints: generating stable and energy efficient forward motion, and coping with changes in the environment or the organism (Sproewitz et al., 2008).

In evolutionary robotics, efficient but straightforward control methods can be found in tables of control sequences (Bongard et al., 2006) and spline-based cyclical patterns (Kormushev et al., 2011). Simple parametric control schemes based on trigonometric functions, like in Koos et al. (2013), have also been successfully applied in a range of experiments.

A more nature-inspired approach exploits central pattern generators (CPGs), which model neural circuitry that outputs cyclic patterns as found in vertebrates (Ijspeert, 2008; Sproewitz et al., 2008). In this case, robot actuators are controlled by the signal generated by coupled synchronized CPGs, allowing synchronized movement. CPGs also require few parameters and provide smooth control transitions when parameters are changed.

Approaches based on CPGs have been successfully applied in several evolutionary robotics and learning contexts, such as for bipedal walking (Reil and Husbands, 2002), salamander-like robots (Crespi and Ijspeert, 2008), and modular robots (Sproewitz et al., 2008; Moeckel et al., 2013).

Clune et al. (2011) used controllers based on more general artificial neural networks to develop controllers for efficient locomotion. They used the HyperNEAT indirect encoding which is based on evolving a Compositional Pattern Producing Network (CPPN) (Stanley and Miikkulainen, 2002; Stanley et al., 2009). We use that very same concept in this experiment, and our main finding entirely relies on CPPN. The CPPN is a network that encodes a function to determine connection weights in the *substrate* artificial neural net that controls the robot. The CPPNs were evolved using NEAT, an evolutionary algorithm specially tailored to the evolution of neural networks (Stanley and Miikkulainen, 2002). However, CPPNs evolved by NEAT do not have to encode neural network connections indirectly, they can, for instance, be used directly as pattern generators for robots (Morse et al., 2013), or be queried for CPG parameters, which is the approach we have applied in our work on robot control (Jelisavcic et al., 2017b).

### 2.3. Lifetime Locomotion Learning

Most of the mentioned research considers the *off-line* development of locomotive controllers, i.e., controller optimization as a separate phase before deployment intending to developing controllers that remain fixed once deployed. Weel et al. (2017) considered *on-line* gait learning, where the controller is adapted to the robot's task environment during deployment. The experiments showed that spline-based controllers with the RL PoWER algorithm provide dynamic autonomous *on-line* gait learning capabilities. Jelisavcic et al. (2016) showed that RL PoWER is very similar to an online (μ+1) evolutionary strategy. The complexity of the problem increases if a directed locomotion is a requirement (Lan et al., 2018).

Note, that the methods for gait development mentioned above are all evolutionary. This may cause some confusion, as we consider them in the role of lifetime learning in an overarching evolutionary process where the morphologies evolve. Thus, we consider systems comprising *two* adaptive processes. At the highest level, the robot morphologies evolve: a new individual implies a unique body that is the result of applying variation operators to its parents' genomes. We have argued that this necessitates a second adaptive process of lifetime learning that operates at a different time-scale to optimize the individual's controller to suit its body and environment. We consider on-line evolution as a suitable technique for this second phase—it can be seen as an instance of reinforcement learning (Haasdijk et al., 2012). So, reiterating: there are two interleaved evolutionary processes: one that adapts morphologies and another that adapts controllers, and the latter implements lifetime learning for the former.

There are two principal options for evolution to exploit lifetime learning: Baldwinian and Lamarckian evolution. The former does not directly store the results of lifetime learning phase, only the resulting fitness values. Lamarckian evolution, by contrast, does explicitly store the locally learned improvements in the individual genomes, so that lifetime learning can directly accelerate the evolutionary process and vice versa (Ackley and Littman, 1994). While this mechanism has mostly not seen as a correct description of biological evolution, some recent research has reported a Lamarckian type of evolution in nature (Dias and Ressler, 2014). Lamarckian learning has also shown to be successful with evolutionary algorithms (Le et al., 2009), and while most lifetime learning experiments in evolutionary robotics have focussed on Baldwinian learning (Nolfi and Floreano, 1999), there have also been reports on efficient Lamarckian approaches (Ruud et al., 2016).

To implement the Lamarckian evolution of morphology and control the robot's genome must encode the robot's controller as well as its morphology. Lifetime learning schemes that directly encode parameters for particular actuators make less sense than indirect encodings: it is difficult or even impossible—e.g., when expression of the morphology is non-deterministic or depends on the environment (Liu and Sen, 2011)—to identify the mapping of controller parameters to actuators in a new morphology where some actuators may no longer occur and new ones have appeared. An indirect encoding scheme such as HyperNEAT would not be hampered in this way: a different layout of actuators would merely imply a change in input values when expressing the genome. Implementations that do encode the robot controllers directly exclude recombination operators and have deterministic morphogenesis and therefore are less susceptible to this issue (Lipson and Pollack, 2000). Several approaches to co-evolution of morphology and control with indirect and coupled body-brain encodings exist, e.g., based on graphs or L-systems (Sims, 1994; Hornby et al., 2003), where the control components are generated along with the morphology.

### 2.4. Triangle of Life Architecture

As explained in Eiben et al. (2012), Eiben and Smith (2015), and Howard et al. (2019) the substrate in which (artificial) evolution takes place does make a difference. The Evolution of Things, e.g., the evolution of robots, is different from the evolution of solutions for a routing problem with a genetic algorithm. Thus, to capture the overall system architecture of robotic systems that evolve in real time and real space we need a framework different from the generic scheme of traditional evolutionary algorithms.

An appropriate system architecture was introduced Eiben et al. (2013) and extensively discussed in Eiben (2014). This framework, named the Triangle of Life, is generic, the only significant assumption is the genotype-phenotype dichotomy that assumes that the robotic organisms that undergo evolution are the phenotypes encoded by their genotypes. This assumption implies two essential system properties. First, that crossover and mutation take place at the genotypic level and second, that there is a mechanism that can produce the phenotype (the robot) corresponding to a given genotype (the code).

**Figure 1:**
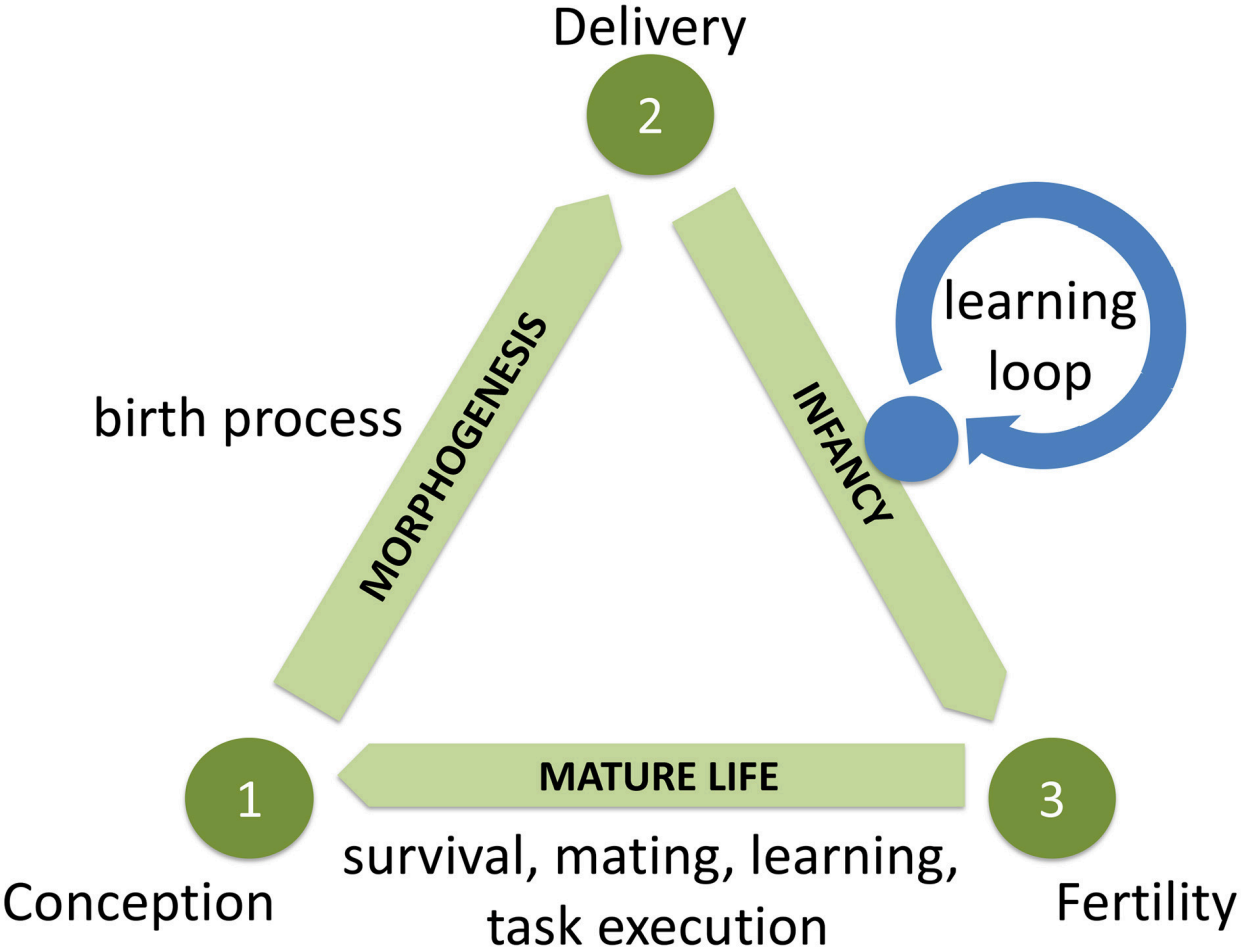
Generic system architecture for robot evolution conceptualized by the Triangle of Life. Note that the *the Learning Loop* in the Infancy stage is not necessarily evolutionary, but in this study we employ an evolutionary algorithm (HyperNEAT) as a learning method.

The resulting framework contains three main components or stages as shown in Figure 1. The pivotal moments that span the triangle and separate the three stages are (1) *Conception*: A new genome is activated, construction of a new robot starts. (2) *Delivery*: Construction of the new robot is completed. (3) *Fertility*: The robot becomes ready to conceive offspring. Thus, the first stage—that can be physically implemented in a so-called Production Center–starts with a new piece of genetic code that is created by mutating or recombining the code of existing robots (the parents) and ends with the delivery of a new robot (the child). The second stage—that can take place in a so-called Training Center— follows the production of a new robot and ends when this robot acquires the basic skills for surviving in the environment and performing user-defined tasks (if any). If the robot successfully passes an application dependent test of these skills, it is declared to be fertile and can enter the third stage. Making fertility depend on some test of quality is an essential design choice meant to prevent the reproduction of inferior robots and the waste of resources. The third stage corresponds to the period of maturity. It is this period when the robot “lives and works” and possibly reproduces. Depending on the given selection mechanism the robot can produce a new genome through recombination and/or mutation and start off a new iteration of the Triangle of Life.

Forced by technological constraints, currently there exist only simulated implementations of a complete Triangle of Life system, such as Weel et al. (2014). Physical implementations are limited to two simplified proof-of-concept studies. Brodbeck et al. (2015) can be perceived as a system with a (semi-)automated Production Center, while Jelisavcic et al. (2017a) demonstrates one life cycle relying heavily on humans in the loop.

## 3. Robot Design

In the following we describe the make-up of our robots distinguishing the bodies and the brains, that is the robot morphologies and the robot controllers.

### 3.1. Morphologies

**Figure 2:**
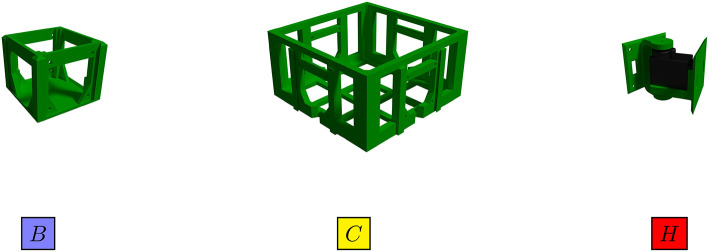
The 3D-printable robot components., **(B)** Fixed brick, **(C)** Core component, and **(H)** Active hinge. These models are used in the simulation, but also could be used for 3D printing and construction of real robots. The blue-, yellow-, and red-colored blocks bellow components illustrate a 2D representation of robots in Figure 15.

The morphologies of the robots in our system are based on the RoboGen framework (Auerbach et al., 2014)^2^. The framework uses a set of seven component types, but we reduced the number to a subset of three 3D-printable components: *fixed brick, core component*, and *active hinge* (Figure 2). The reasoning for using the subset of components is to simplify the analysis of the evolutionary process. The two-part model defines each of these components used in the simulation: a detailed mesh suitable for visualization and 3D-printing and a set of geometric primitives that define the components mass distribution and a contact surface.

Following the standard RoboGen specification, each component is described with the *type of the component* it represents, its *name, orientation*, possible *parametric values*, and *children slots* to which neighboring components are connected^3^. For making the evolution of morphologies more flexible, the RoboGen specification was extended to define the *attachment slot* to its parent component^4^. To simplify the identification of the heredity of each robot's morphology the specification was supported with a color-coding for each component. This allows the morphological traits that a robot inherits to be easily attributed to either parent by matching colors.

**Figure 3:**
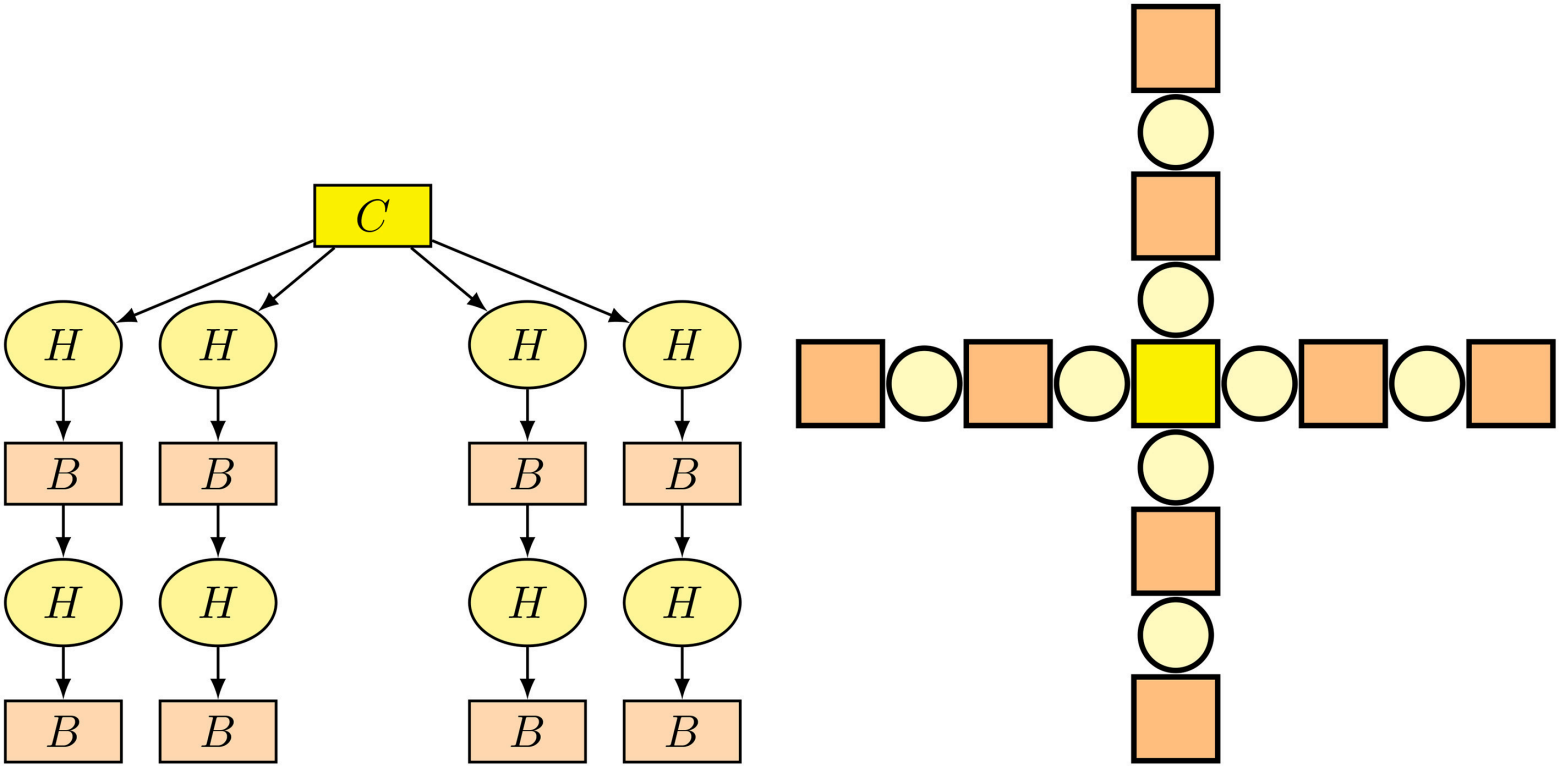
An illustration of a robot's genome **(left)** for one of the possible morphologies and the outcome of assembled morphology **(right)**. On the genome representation, a *fixed brick* module is designated with *B*, a *core component* with *C*, and an *active hinge* with *H*.

Robots are genetically encoded by a tree-based representation where each node represents one building block of the robot (i.e., a RoboGen module) and edges between nodes represent connections in between. An example of a genome's tree structure for one of the possible morphologies is shown in Figure 3. The construction of a robot in this representation begins with the root node, which by definition always represents the essential core component. The robot body is then constructed by traversing the tree edges and attaching the components represented by child nodes to the current component at the specified slot positions and orientations. Details about recombinations and mutations are described in subsection 4.1.

### 3.2. Controllers

The controller system for the robot locomotion consists of two main components —a *CPG controller structure* derived from a robot's body structure, and *weights of CPG connections* derived as outputs of a CPPN network. In these controllers, sensory feedback is omitted, because an open-loop controller can solve the given task (gait learning). Including sensory feedback is a possible augmentation to the CPG structure. However, the primary goal is to investigate effects of the specific evolutionary setups on body development. Adding an extra dimension in form of the feedback loop to the system would make it harder to distinguish an effect of the system from the sensory noise.

**Figure 4:**
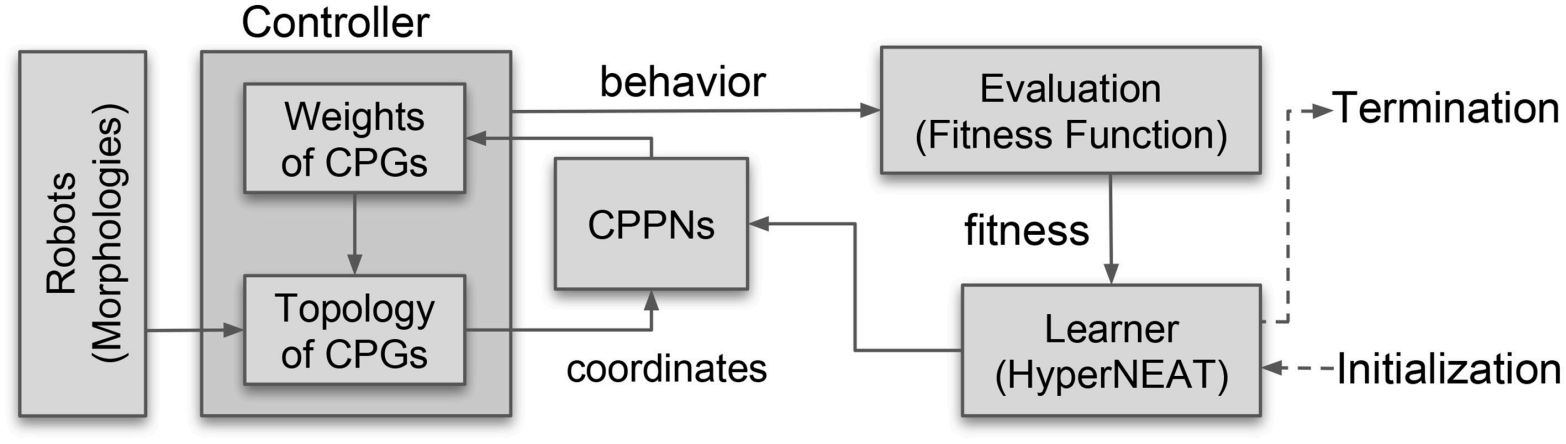
The overall architecture of the learning system. The learning method is implemented by an evolutionary algorithm (HyperNEAT). It evolves the CPPN that defines the connection weights of the CPG-based controller whose topology is based on the morphology of the given robot.

Figure 4 depicts the resulting architecture. The CPG is firmly grounded in the morphology of a given robot (explained below). The part that can be transferred between different robots is the CPPN. This is very important as it enables us to transfer controllers between different bodies.

**Figure 5:**
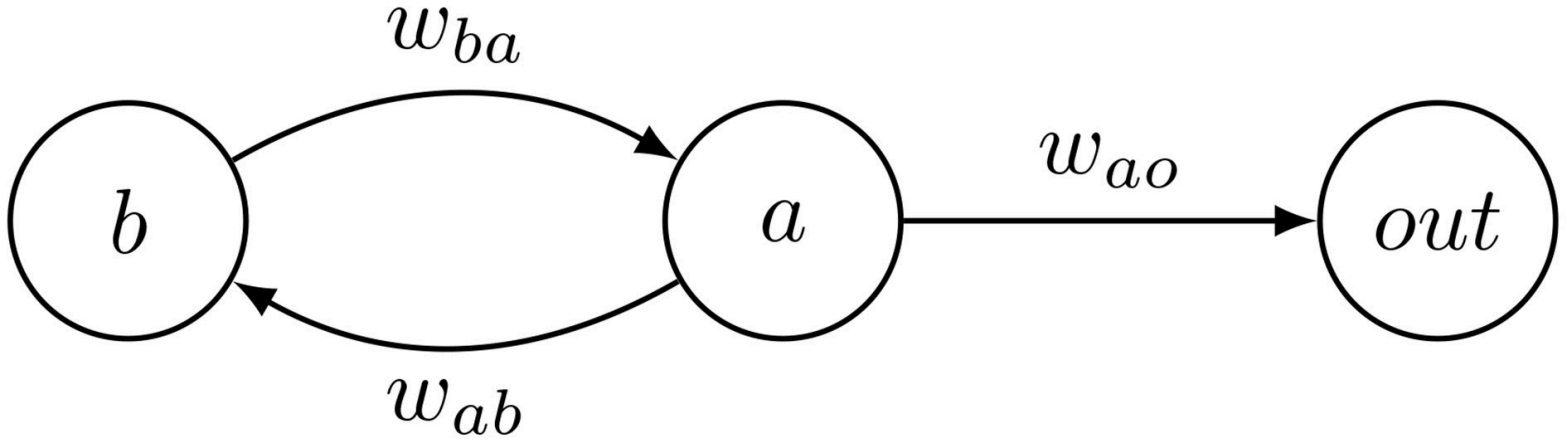
A differential oscillator with output node as used in the CPG controller.

The main components of the CPG controllers are differential oscillators. One oscillator is defined for each active hinge. Each oscillator is defined by two neurons that are recursively connected as shown in Figure 5.

These generate oscillatory patterns by calculating their activation levels *a* and *b* according to the following differential equation:

$$\begin{array}{l} ȧ=w_{ba}y+{\text{bias}}_{a}\\ ḃ=w_{ab}x+{\text{bias}}_{b} \end{array}$$

with *w*_*ab*_ and *w*_*ba*_ denoting the weights of the connections between the neurons; *bias*_*a*_ and *bias*_*b*_ are parameters of the neurons. If *w*_*ba*_ and *w*_*ab*_ have different signs the activation of the neurons *a* and *b* is periodic and bounded.

An oscillator's *x* node is connected to a linear output neuron that in turn connects to the robot's active hinge. Output neurons use the following activation function:

$$\begin{array}{l} f\left(a\right)=\left(w_{ao}\cdot a-\text{bias}\right)\cdot \text{gain}. \end{array}$$

with *a*, the activation level from the oscillator, *w*_*ao*_, the weight of the connection between oscillator and output node and *bias* and *gain* parameters. Each active joint in the robot body is associated with an oscillator and connected to it through an output neuron that determines the joint's angle.

**Figure 6:**
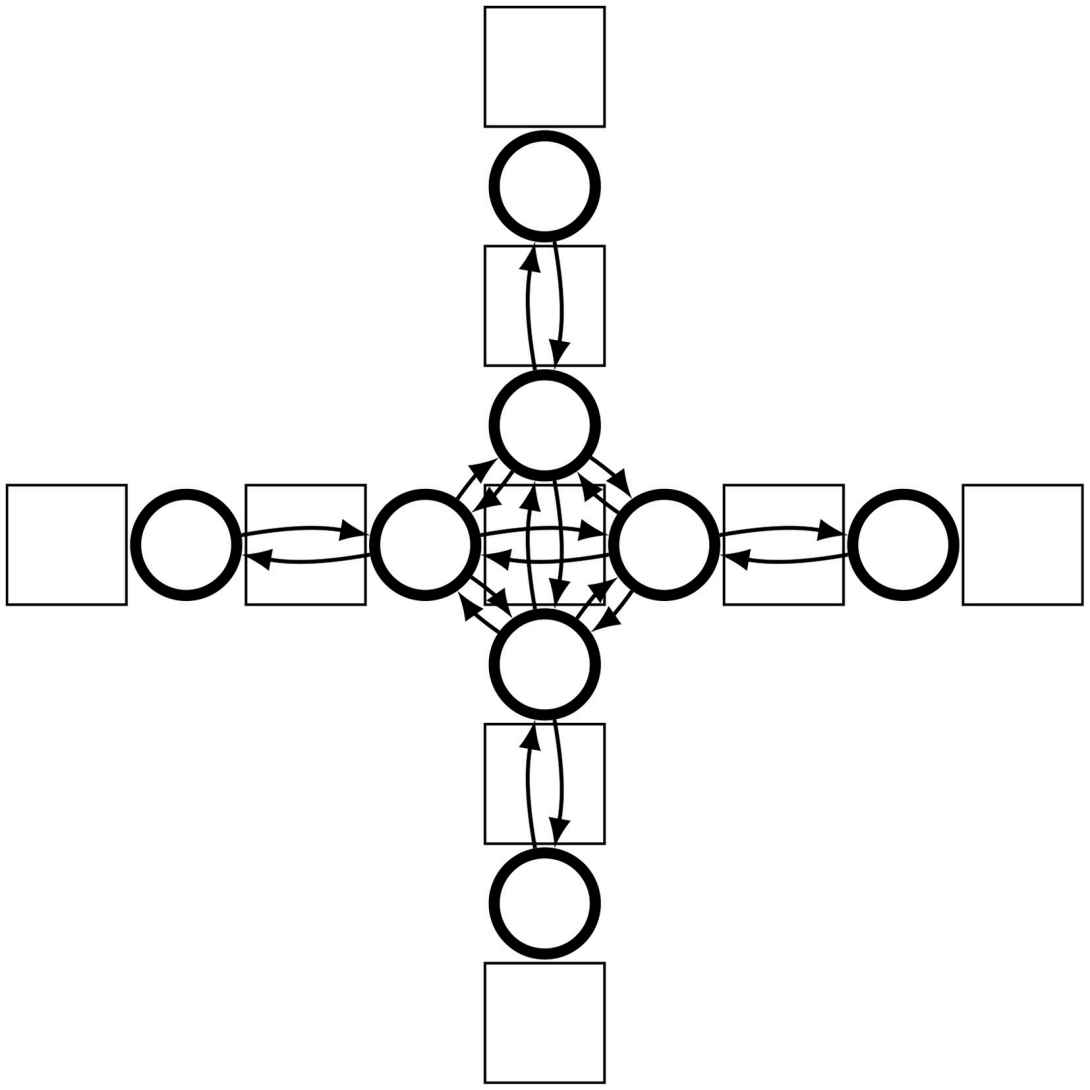
Schematic view of the CPG network generated for one of the possible morphologies. The rectangular shapes indicate passive body parts, the circles show active hinges, each with their own differential oscillator, and the arrows indicate the connections between the oscillators.

The oscillators of neighboring hinges (i.e., hinges separated by a single component) are interconnected through weighted connections between their *a* neurons. This results in a chain-like neural network of differential oscillators that extends across the robot body, as illustrated in Figure 6.

Like a neural network, a CPPN is a network of mathematical functions with weighted connections. Unlike neural networks, the network can contain a variety of activation functions including *sine, cosine, Gaussian*, and *sigmoid*. The CPPNs have six inputs denoting the coordinates of a source and a target node when querying connection weights or just the position of one node when obtaining node parameters with the other three inputs being initialized as zero. The CPPNs have three outputs: the weight of the connection from source to target as well as the bias and gain values when calculating parameters for a node. To determine the weight of a connection in the CPG network that controls the robot (the substrate), the coordinates of the two substrate nodes are fed into the CPPN which then returns the connection weight (Stanley, 2007). In order to obtain the parameters of a node, the coordinates of that node and the all-zero vector (instead of a coordinate of the other node) are used as inputs. This way enables us to select either a connection between two nodes, or a specific node itself.

**Figure 7:**
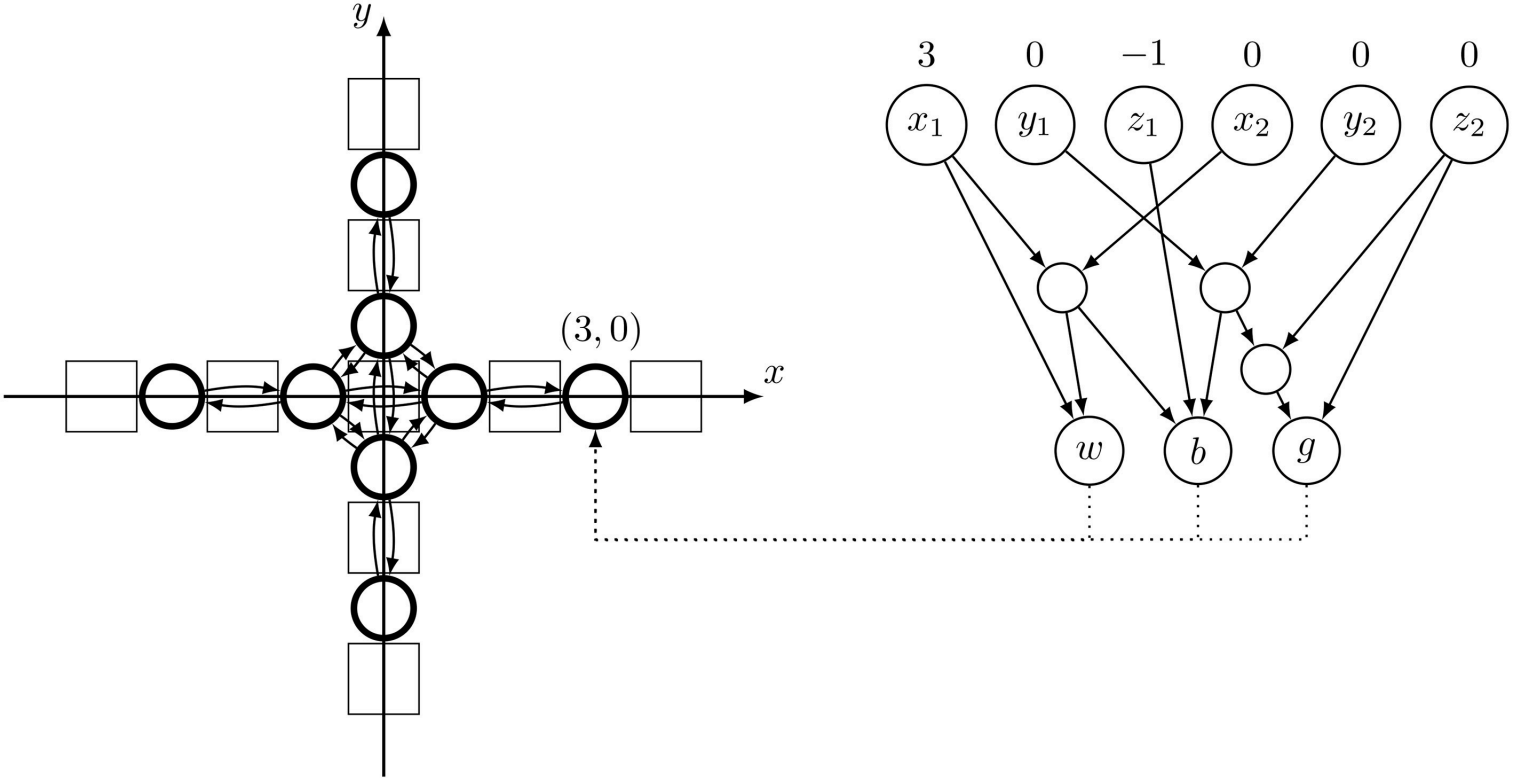
Example of the lifetime process of applying the proper weights from a CPPN network onto a CPG neuron. The arrow is pointing to a neuron within a differential oscillator with coordinates (*x, y*) = (3, 0) and *z* = −1 for the *b* node (see Figure 5).

Further on, we will explain how the controllers' genotypes (CPPN) and phenotypes (CPG) are connected. The important piece of the puzzle is the Cartesian coordinate system, universal to any body structure with a referent (0, 0) coordinate positioned in the core component. Based on this, the CPG nodes are positioned in a three-dimensional hyperspace. The hyperspace consists of a planar space with *x* and *y* coordinates that position a differential oscillator (i.e., an active joint) and a third coordinate *z* which define a position of a neuron in the oscillator. Such *modular differentiation* allows specialization of the active hinge‘s movements depending on its relative position in the robot. The hinge coordinates are obtained from a top-down view of the robot body. Thus, two coordinates of a node in the CPG controller correspond with the relative position of the active hinge it is associated. The third coordinate depends on the role of the node in the CPG network: output nodes have a value of 0, and differential nodes have values of 1 for *a* and −1 for *b* nodes. As illustrated in Figure 7, differential CPG is defined based on a robot's body structure, and weights between connections are defined by applying output values of evolved CPPN network on it.

Example for the process of applying parameters to a specific neuron in a CPG network is illustrated in Figure 7. On the CPG structure, the coordinates of each active hinge are illustrated. In order to define the values for the *b* node on the coordinate (3, 0), we designate (*x*_1_, *y*_1_) = (3, 0) and *z*_1_ = −1. To mark that we are querying for node values and not weight of a connection the second part of the CPPN input (i.e., the destination node) is marked as (*x*_2_, *y*_2_, *z*_2_) = (0, 0, 0).

Based on values fed into a CPG network, a different output pattern is produced for every actuator resulting in a different locomotion behavior. The given learning algorithm then iteratively improves the CPPN in order to optimize the connection weights, the node biases, and the gain levels of the output nodes produced by it. Details about our learning algorithm are presented in the next section.

## 4. Evolutionary and Learning Methods

The general system architecture as captured by the Triangle of Life consists of two loops: the evolutionary loop that spans the triangle and the learning loop within the Infancy stage, see Figure 1. The evolutionary process affects both the bodies and the brains, while the learning algorithm only works on the brains. As mentioned before, in this study we decided to use HyperNEAT for gait learning (Stanley and Miikkulainen, 2002). This implies that our learning method is an evolutionary algorithm. Consequently, we have two evolutionary processes. The outer evolutionary process (with a Darwinian and a Lamarckian version) works on the morphologies and the controllers, and the inner evolutionary process (HyperNEAT) works on the controllers within the Infancy stage.

### 4.1. Evolution of Morphologies

The evolutionary process of morphologies uses a direct encoding and is relatively straightforward using recombination and mutation operators defined in RoboGen. As described in subsection 3.1, the morphologies of the robots are represented as tree structures where every node represents one component. Therefore, we can apply the recombination and mutation operators that are well-established in genetic programming (Banzhaf et al., 1998). The recombination of parent genomes is implemented as random subtree exchange: a random node from parent *A* is selected and replaced with a random subtree of parent *B* if only the exchange does not violate the constraint of intersecting body parts. In order to limit robot complexity, a new part is added with a probability proportional to the number of parts that are expected to have been removed by subtree removal, minus the number of parts expected to have been added by subtree duplication. The new part is randomly generated and attached to a random free slot on the tree to produce the final robot. To keep the complexity of the robot morphologies within bounds we require that each robot must consist of at least five and no more than 50 modules. The parameters of these operations are shown in Appendix 3. The mutation operator is applied after each crossover directly to the generated offspring and replaces a randomly selected subtree with a randomly generated tree. The probability of mutation is set to 0.05.

By design, we are using non-overlapping generations. Hence, after evaluating the current population of 20 robots we need to select 20 pairs of parents to produce the next generation. To this end, we use standard binary tournaments. To obtain two parents for crossover we perform two tournaments and for each tournament we select two potential parents randomly. These are compared and the one with the highest fitness (e.g., highest speed) wins the tournament and becomes a parent.

### 4.2. Learning of Controllers

We use HyperNEAT for learning the morphology independent parts of the robot controllers. As described in section 3.2, this means evolving CPPNs for setting the CPG parameters. We chose to apply a population size of 10 CPPNs, which means that each robot has 10 controllers on board that can be activated and tested one by one. The method to fill the initial controller population after the ‘birth' of a new robot depends on the Darwinian or Lamarckian nature of the algorithm for controller evolution. These details are explained in section 4.3. A full overview of HyperNEAT parameters is given in Appendix 3.

HyperNEAT requires bookkeeping of the complexification process that occurs by adding and removing nodes and connections. This is implemented employing innovation numbers that uniquely identify inserted material. The innovation number introduced by the same mutation of the network is identical across different networks for nodes and connections. This solution combines two interacting ideas. Firstly, the introduction of a crossover operator which randomly can choose from which parent a neuron or connection should be inherited if it is present in both. This can be useful since neurons or connections in a topological context should fulfill a similar purpose and therefore swapping them will not give a completely different network, only a slightly changed one. Secondly, the usage of speciation, which groups networks with similar neuron and connection parameters as well as similar topologies. This can easily be inferred due to the usage of innovation numbers, thus ensuring that mostly similar networks are crossed over. Together these ideas help reduce the likelihood of introduction of dysfunctional networks through crossover.

When combining CPPNs from two separate HyperNEAT runs (i.e., the learning processes on two different robot morphologies), the innovation numbers must be updated so that no conflicts occur. Offsetting the innovation numbers from one of the two parents proved a convenient method to achieve this, allowing the CPPNs from both parents to be (re)combined in the offspring's population.

### 4.3. Darwinian and Lamarckian Evolution of Controllers

Darwinian evolution as observed in nature works through an inheritance mechanism that cannot propagate traits of an organism to its offspring that are learned by experience. In contrast, the idea of Lamarckism assumes that an organism can pass traits acquired over its lifetime to its offspring. In evolutionary robot systems, the inheritance of controllers can be implemented in a Darwinian or in a Lamarckian way, depending on the preferences of the system designer. In both cases, a “newborn” robot *A* starts its lifetime and the infant learning process of the Triangle of Life scheme with the initial controller inherited from its parents. At the end of this process robot *A* will contain a learned controller and its fitness is determined by how well this works. If the robot is fit and gets selected for reproduction, then it will propagate its morphological and controller properties to its offspring. The difference between a Darwinian and a Lamarckian system is grounded in the different methods for propagating parental controllers. If the offspring, robot *B*, is seeded with the initial controller robot *A* was born with, then we have a Darwinian system. If *A* passes on its learned controller to *B* then the system is Lamarckian.

**Figure 8:**
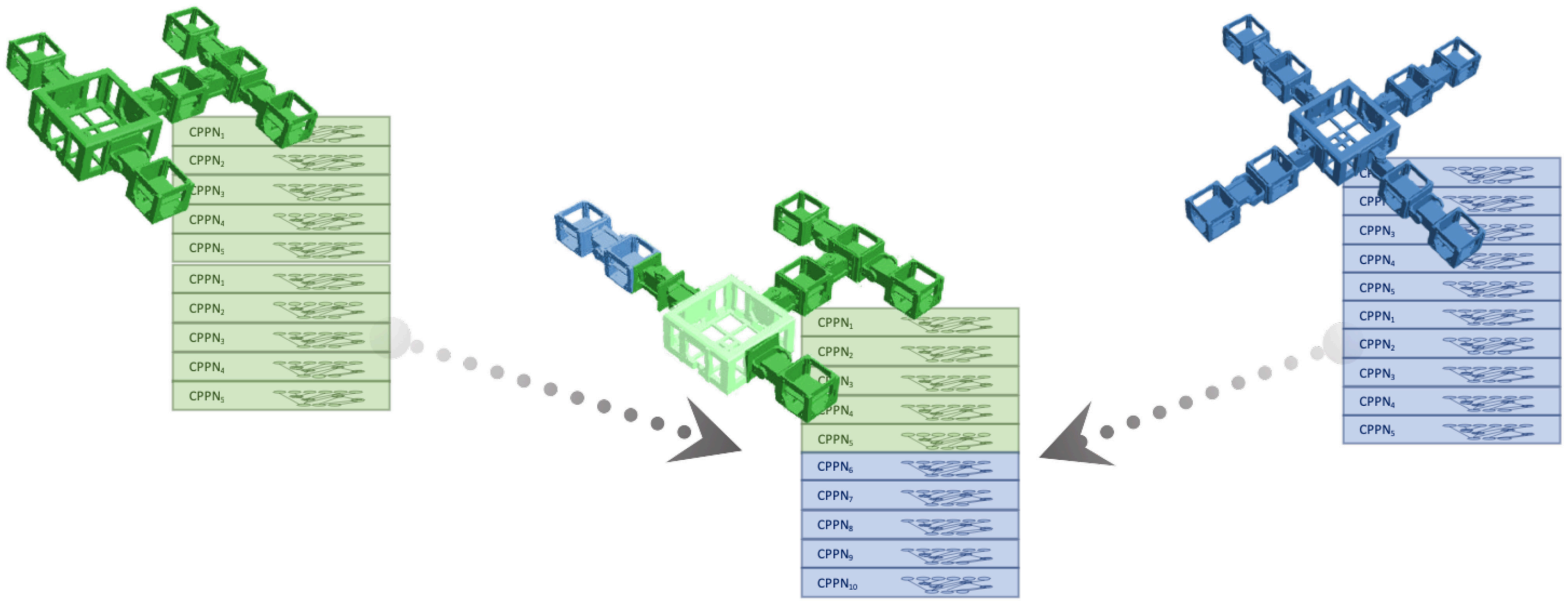
Illustration of transferring controllers from parent robots to their offspring. The best five controllers from each robot are transferred to their child, where they form the initial population of the HyperNEAT algorithm for gait learning.

As explained in the previous section, in this study we use an evolutionary algorithm (HyperNEAT) with population size 10 as a learning method. This means that each “newborn” robot needs to be initialized with 10 controllers. Exploiting the fact that the CPPNs are morphology independent, i.e., that any CPPN can be used in any robot, the inheritance of controllers can be done in a simple manner, including five controllers from one parent and five from the other parent in the child. For obvious reasons, each parent passes on the best five of its controllers to the offspring. This method is illustrated in Figure 8.

## 5. Experiments

Implementing an evolutionary robot system as outlined above in real hardware is not possible with the current technology. Therefore, we conduct our experiments with the Revolve simulator, cf. Hupkes et al. (2018)^5^. Technically, Revolve is a wrapper around Gazebo, a general-purpose robotic simulator based on the ODE physics engine, that manages insertion, deletion, and production of robots in a simulated environment. The settings of the simulation parameters are presented in Appendix 3.

The experiments are executed on five different *lineages*, where a lineage is specified by a randomly generated initial population of robots. These five initial populations are the starting points for the thereafter separate Darwinian and Lamarckian evolutionary runs, leading to different populations of morphologies. In all cases, the population size is 20 and the run is terminated after 10 generations.

The initial robot populations are created by randomly generating 20 morphologies that contain at least five active joints and no more than 50 components all together. Furthermore, it is required that it is physically feasible to construct the given robot. Consecutive generations are produced by the usual selection-reproduction cycle in a non-overlapping fashion. Thus, the total number of robots in an evolutionary lineage is 200. The evolutionary operators crossover, mutation, and selection work as explained in section 4.1 and Appendix 3. As for fitness, we use speed. For any given controller the speed equals *m*/*s*, where *s* is the duration of the evaluation period (30 s as shown in Appendix 3) and *m* is the straight line distance the robot covered in this period. For a robot as a whole, the fitness is the maximal speed, achieved by the end of its learning period.

Regarding the controllers of the robots, we use an evolutionary algorithm, HyperNEAT, as a learning method with settings described in Appendix 3. We set the population size in HyperNEAT at 10 and the ‘heads' of the initial robot populations of the five lineages are filled with CPPNs consisting of randomly initialized networks that only contain the input and output neurons and connections from every input to every output neuron with randomly initialized weights and neuron parameters. A HyperNEAT run—that is, a gait learning process of the given robot body—can be terminated after 10 or 100 or 1,000 evaluations. This means 1, 10, or 100 non-overlapping generations^6^.

To compare the Lamarckian and the Darwinian systems we monitor the average performance (i.e., speed) of the robots during each run. Furthermore, we carry out another analysis regarding the amount of parental material in the controllers. To this end, note that the 10 CPPNs of a “newborn” robot are all inherited from its parents, in the Darwininan as well as in the Lamarckian setup. Hence, the learning period—a run of HyperNEAT—starts with 100% of parental CPPN genetic material. During the run of HyperNEAT the CPPNs are augmented with new neurons and connections, thus the ratio decreases. The ratio of parental material that is present in the CPPNs' structures at the end of a learning period is called parental retention.

## 6. Results

**Figure 9:**
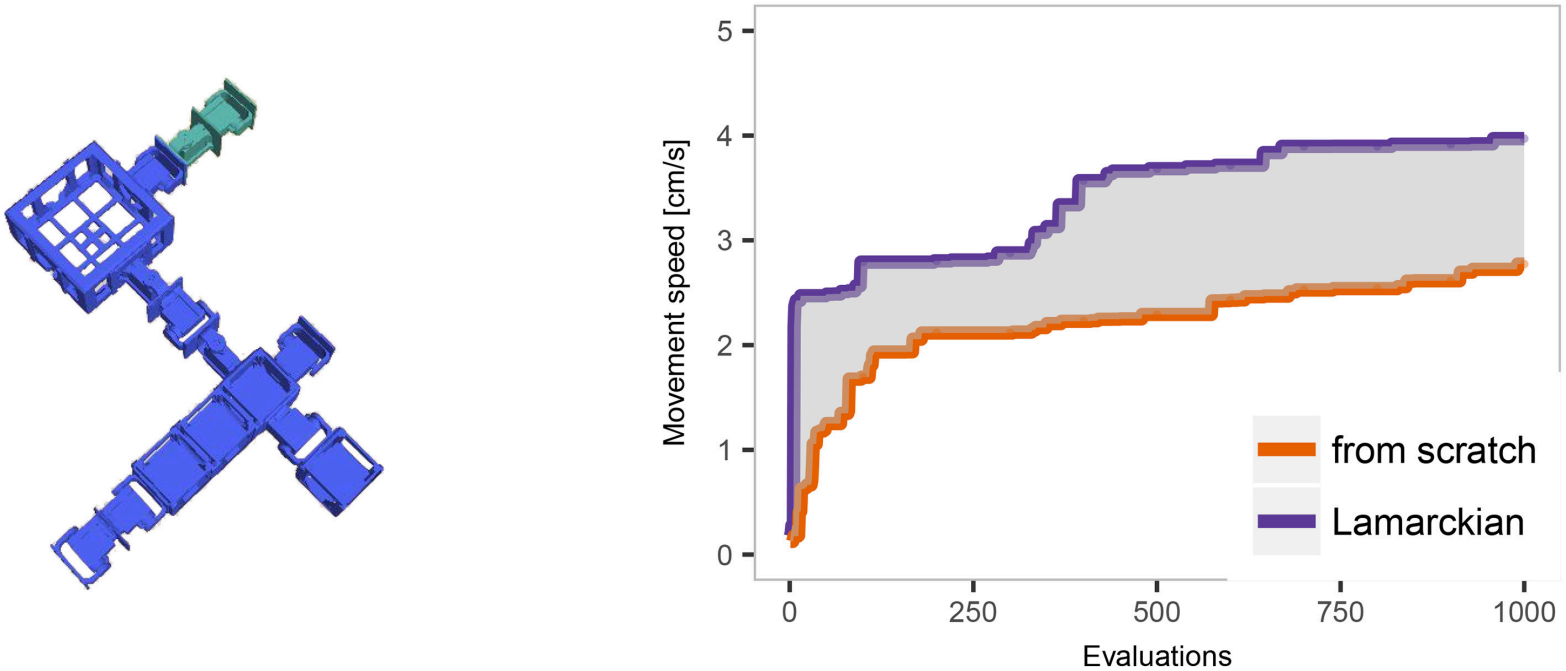
Example of an evolved robot from lineage 3, generation 2. The colors of its modules disclose their origins, blue and green modules come from different parents. The curves on the right hand side show the development of speed of this robot during the learning period starting from scratch or based on Lamarckian inheritance.

Before presenting the main results let us give a simple illustration of the effect of Lamarckism in our system. For this purpose, we take an evolved robot (shown on the left of Figure 9) and plot the development of speed during a lifetime learning process starting with a) 10 randomly initialized CPPNs and b) 10 CPPNs inherited from its parents after they finished learning. These curves are shown on the right hand side of Figure 9. The locomotion strategies employed at different stages of the learning process can be seen in a Supplementary Video^7^. The differences in performance are representative for many of the robots in our experiments, but there are also a few morphologies where learning from scratch outperforms learning from inherited material. This warrants further analysis of the relations between performance, morphologies, and brains, as detailed in the following sections.

### 6.1. Performance Differences

**Figure 10:**
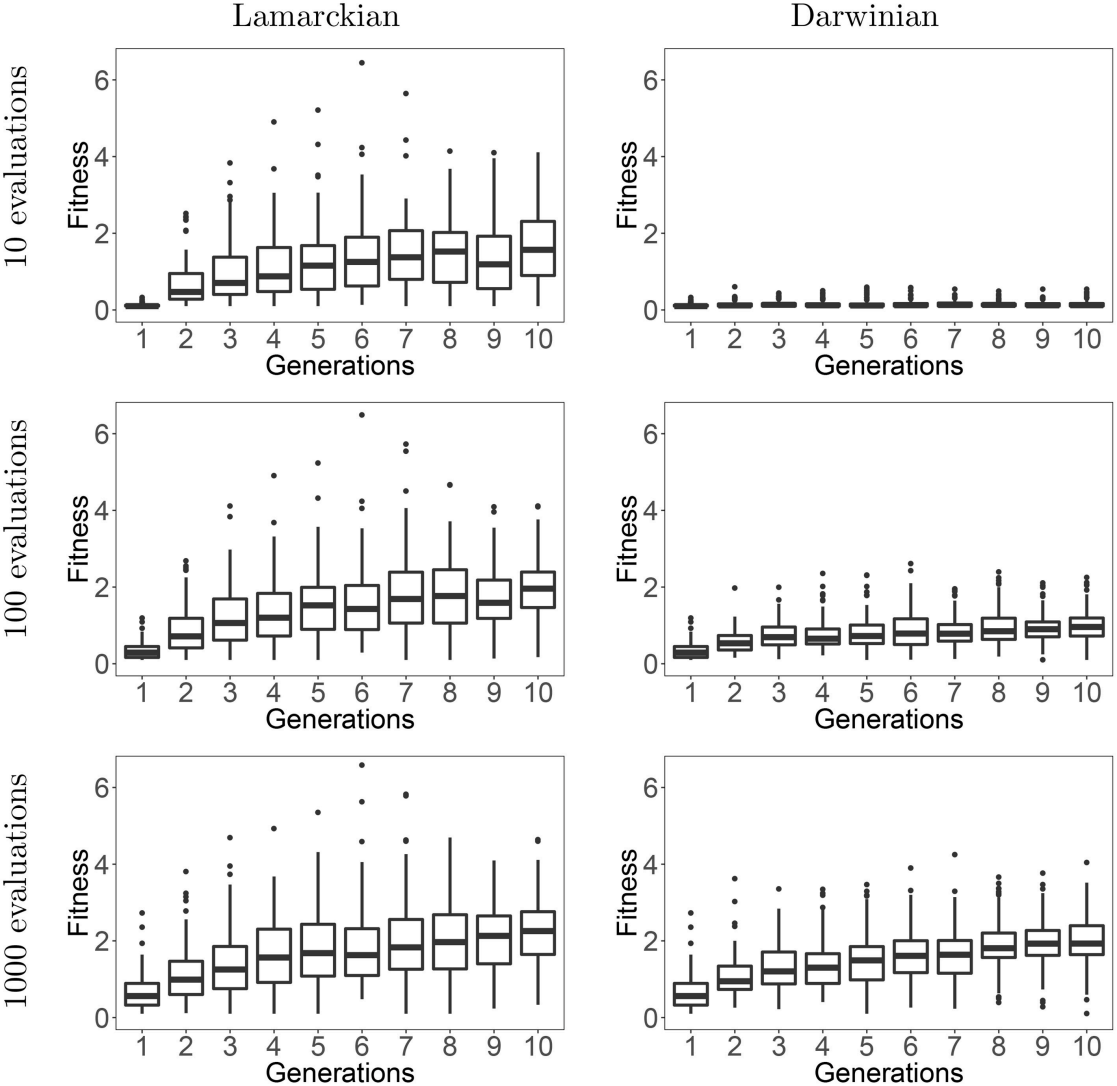
Development of fitness over the course of evolution for Lamarckian (left column) and Darwinian evolution (right column) based on a low, medium, or high budget for the lifetime learning method, i.e., 10/100/1,000 CPPN evaluations within the HyperNEAT algorithm.

To answer our first research question we compare the Lamarckian and the Darwinian evolutionary approaches. For this purpose, we merge the data of all five lineages and plot the fitness values in each robot generation in Figure 10. The plots the show development of fitness over the course of evolution for both approaches with three different learning budgets: low (10 CPPN evaluations, 1 generation in HyperNEAT), medium (100 CPPN evaluations, 10 generations in HyperNEAT), and high (1,000 CPPN evaluations, 100 generations in HyperNEAT).

Looking at the plots, we can observe the superiority of the Lamarckian approach. The differences in performances are most prominent for a low and a medium learning budget, when using 1,000 evaluations for learning the differences are smaller. This indicates that the Darwinian approach can eventually catch up with the Lamarckian system, but only at a large expense in the number of evaluations. This can be consequence of converging to a nearly maximal speed for the given morphology. Phrasing it differently, if the resources are constrained, the Lamarckian approach is advisable. This is especially important to consider in a real-world evolution scenario (in the future).

**Figure 11:**
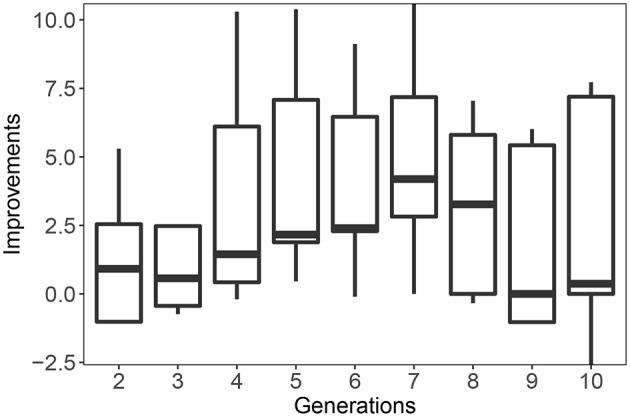
Improvement of Lamarckian over Darwinian evolution per generation. Improvement is defined as the difference in average performance of all individuals of all lineages in the given generation. A positive number means that the Lamarckian approach is better.

We can shed further light on the evolutionary dynamics by looking at the difference in fitness between the Lamarckian and Darwinian evolutionary process per generation. That is, we consider the difference in fitness values from the start to the end of the lifetime learning process, like in Figure 9, but in this case the fitnesses are based on population averages We plot these data in Figure 11 for the case of using a high learning budget. In the early generations, the Lamarckian approach is clearly better, but in the last generations the Darwinian setup reaches the performance of the Lamarckian one. The overall conclusion is similar to that made after analyzing Figure 10, if the resources are constrained (here: we only have time for a few generations), then the Lamarckian approach is preferable.

### 6.2. Morphological Analysis

To answer our second research question, we inspect the evolved morphologies in two different ways. First, we look at the genotypic differences between parents and offspring using the Zhang-Sasha method for the tree-edit distances, cf. Zhang and Shasha (1989). Second, we consider the phenotypes and characterize the forms of the robots through seven different morphological descriptors, *size, symmetry, proportion, number of active joints, coverage, length of limbs, number of limbs*, after Miras et al. (2018). Details are given in Appendix 2.

**Figure 12:**
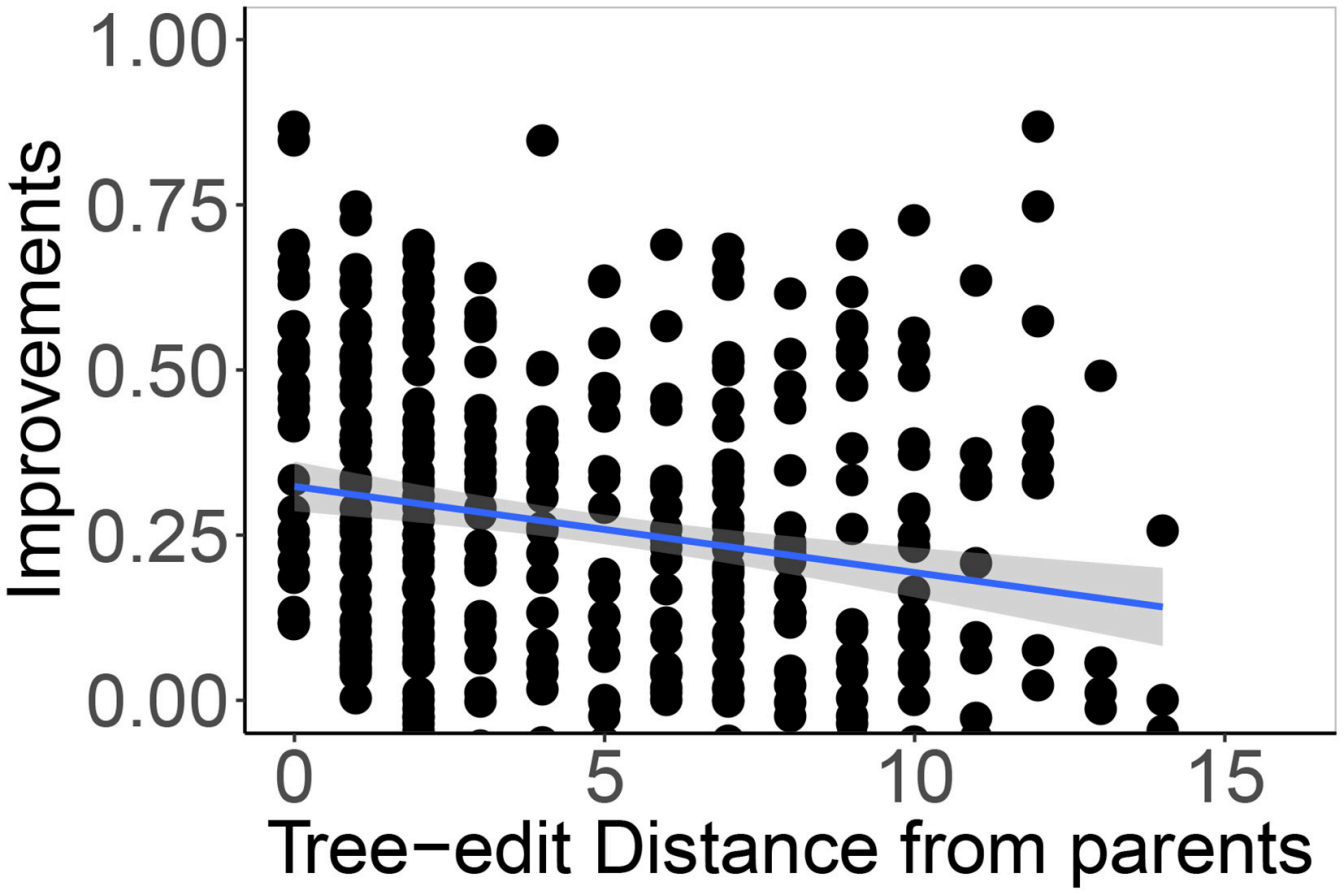
Correlation of normalized tree-edit distances between a parent and its offspring and improvements in performance the offspring shows. The data points are individual performance differences over nine generations (the second up to tenth) in five generated lineages, aggregated. For correlation we use 90% confidence level interval for predictions from the linear model.

The correlation of the normalized tree-edit distances between a parent and its offspring and the advantage of Lamarckian inheritance compared to learning from scratch is exhibited in Figure 12. The data show a trend: the smaller the difference of a robot from its parents, the higher is the benefit of inheriting the controller the parent learned, instead of learning the controller from a random start. Intuitively this makes sense because it stands to reason that the controllers that are optimized for a specific morphology are more useful for a similar morphology than for a different one.

**Figure 13:**
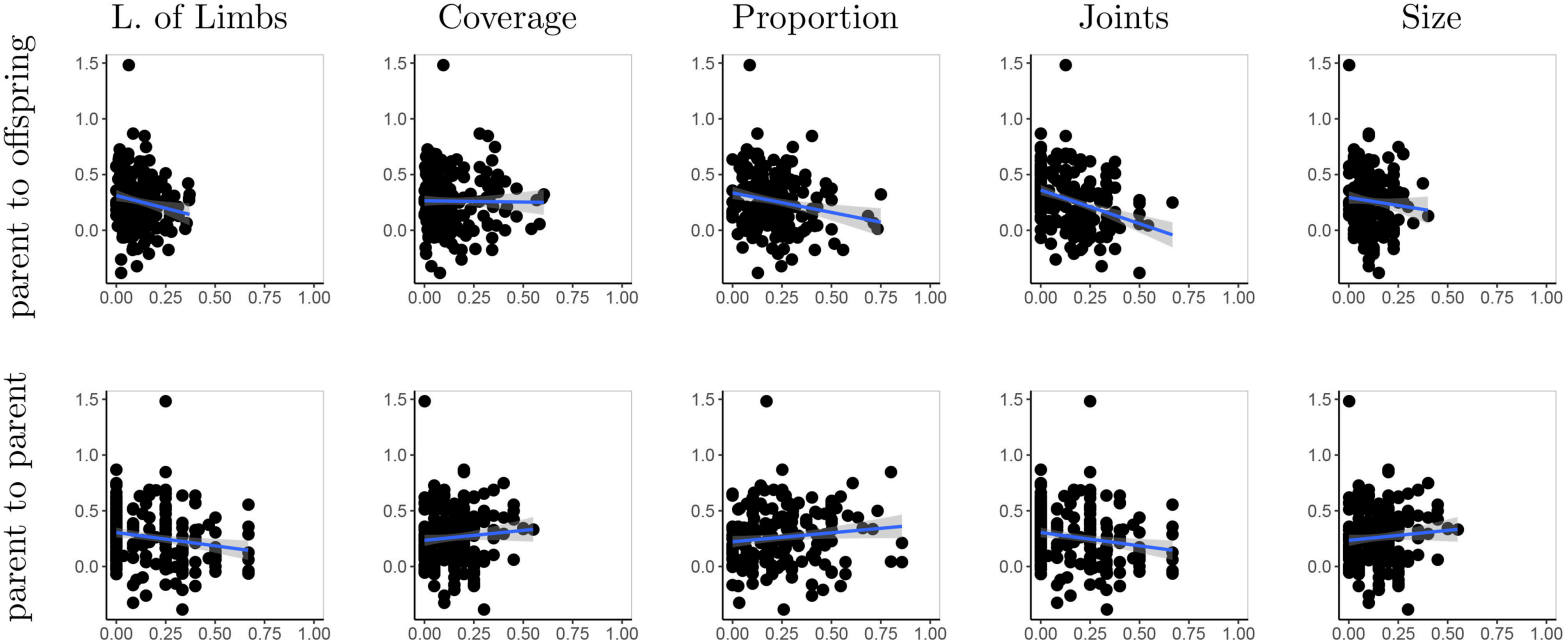
Averaged variance of four morphological descriptors between parents and their offspring (top row), and averaged variance between parents (bottom row) correlated with the observed improvements. For correlation we use 90% confidence level interval for predictions from the linear model.

Using the morphological descriptors we can define the difference between two robots (e.g., two parents or a parent and a child) based on their phenotypes. Because all descriptors have numerical values, we can simply apply the numerical difference here. With the sub-questions of our second research question in mind we can try to correlate the advantage of Lamarckian evolution to a) the difference between a robot and its parents, and b) the difference between the parents. The data corresponding to option a) are shown in the top row of Figure 13 for the five morphological descriptors where a trend could be observed. These trends are similar to the one regarding the genotypic distance: the benefit of Lamarckian evolution is higher if the difference of a robot to its parents is smaller. The bottom row of Figure 13 show the trends when considering the difference between the parents. The trend is not so clear in this case and this is natural: the only reason why there would be a similar trend is because two similar parents have a greater potential to create a robot that's similar to them and thus transfer controllers that are adapted for their morphologies. The correlations for all five measures are verified in Table 1 with Spearman's correlation.

**Table 1:** Coefficient values of Spearman's rank correlation of improvement to tree-edit distances and morphological descriptors (parent to offspring).

	**coef**.	***p*-value**
Tree-edit Dist.	−0.2	1.6 × 10^−11^
L. of Limbs	−0.27	1.9 × 10^−4^
Coverage	−0.29	8.56 × 10^−5^
Joints	−0.3	4.38 × 10^−5^
Proportion	−0.2	5.2 × 10^−3^
Size	−0.23	1.7 × 10^−3^

### 6.3. Morphological Attractors

**Figure 14:**
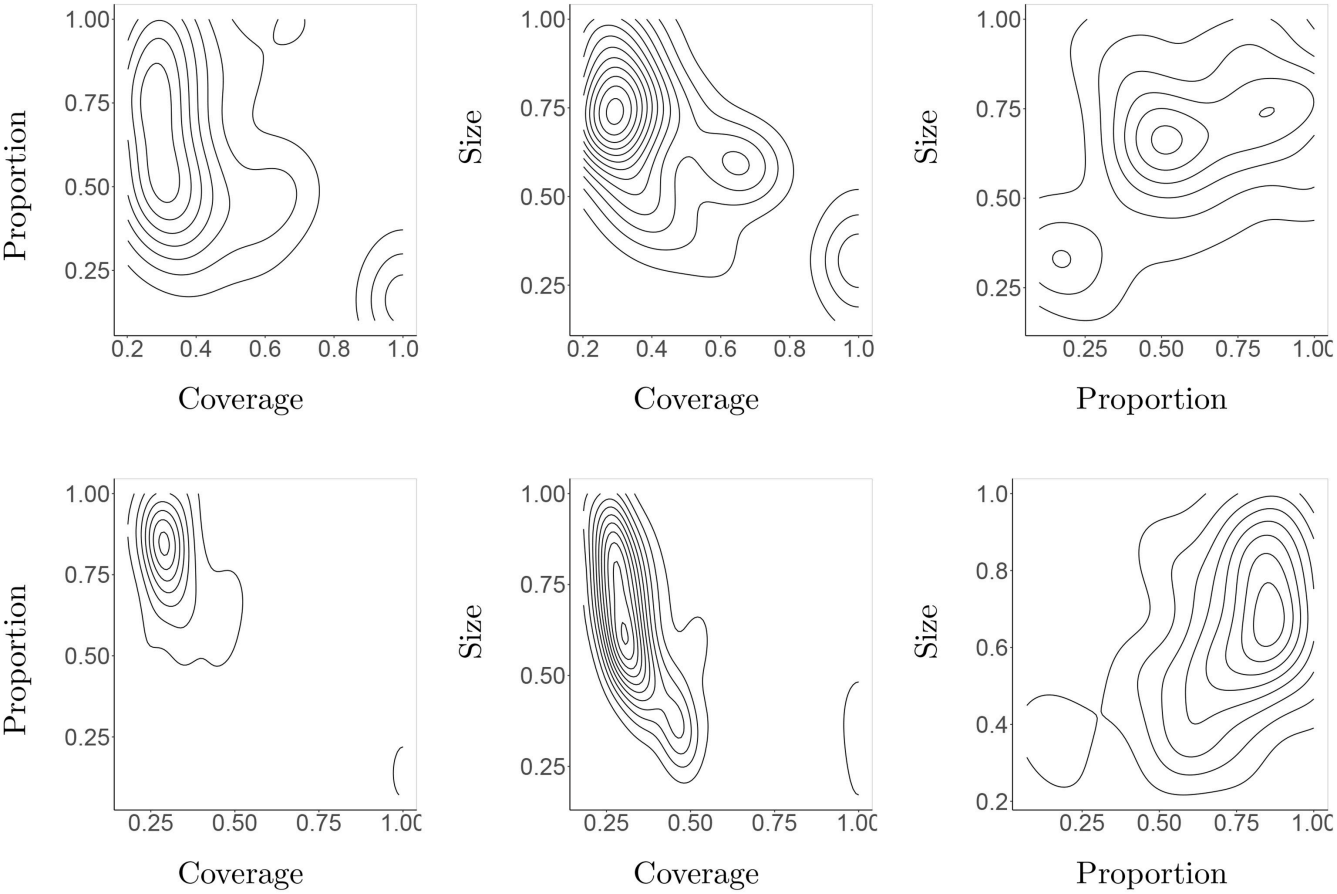
Density areas for the three most prominent morphological descriptors in the 10th generation of both Darwinian **(top row)** and Lamarckian **(bottom row)** regimes.

Based on morphological descriptors we can obtain new insights by creating density plots in the multi-dimensional space they span. We find this a useful tool that gives insights into our third question. For reasons of printability, we selected a few pairs of descriptors and created contour plots in the corresponding 2-dimensional space showing the regions with the highest density, that is, with a relatively high number of evolved robots. Now the question is if these areas of attraction differ for Darwinian and Lamarckian evolutionary setups. The most interesting density plots are presented in Figure 14 and the whole set in Figure S3 in the Appendix. We can see that the Lamarckian robots are more consistent, while the Darwinian robots tend to scatter more over the morphological search space. This observation is interesting as it indicates that treating the (learning of) controllers differently changes the development of morphologies too.

**Figure 15:**
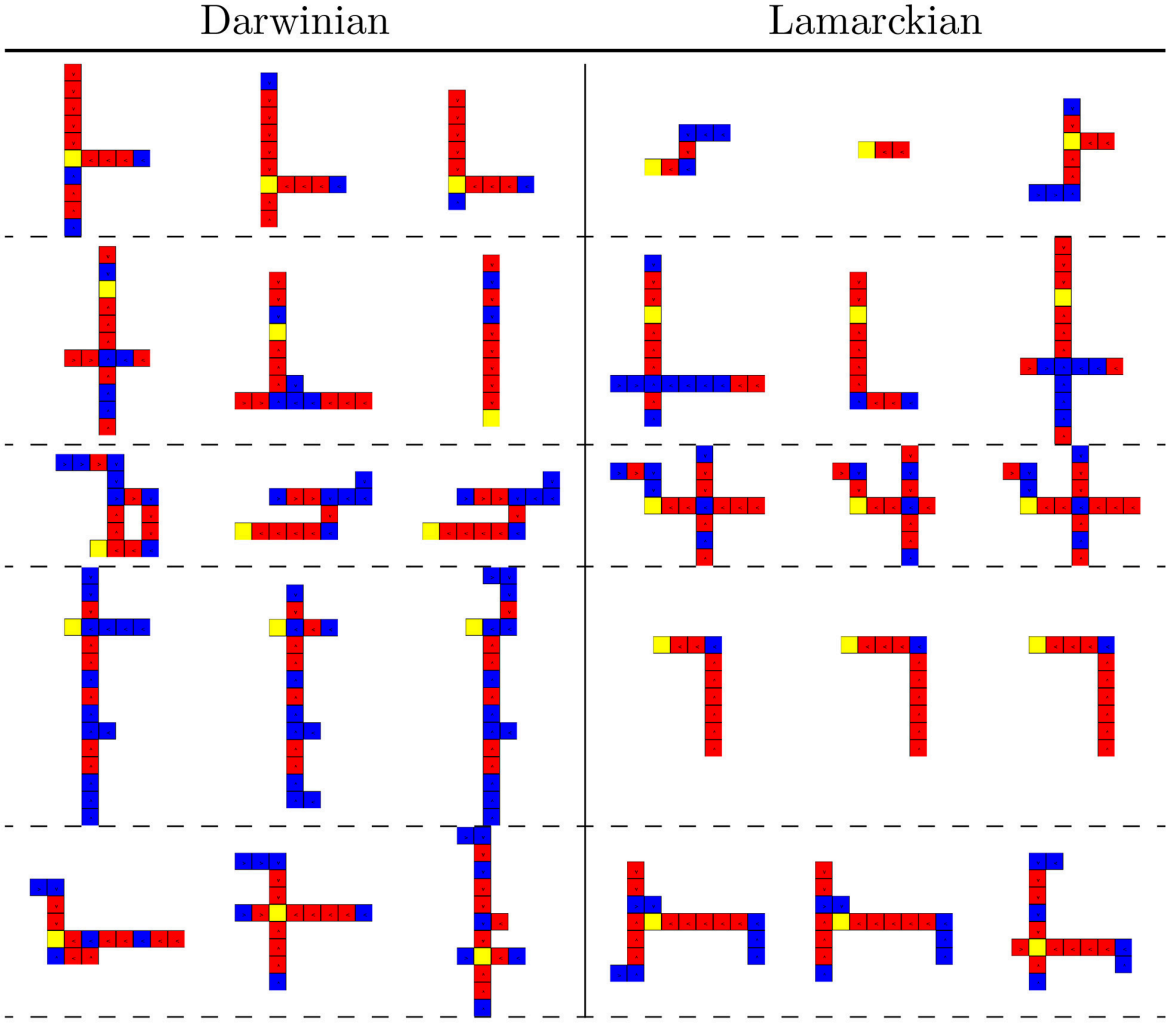
The best three morphologies of the final (10th) generations in all 5 lineages in both scenarios. The Darwinian setup **(left)** develops predominantly I- and L-shaped robots. The Lamarckian setup **(right)** develops X- and T-shaped robots.

The morphologies of the evolved robots can be visually inspected as well. In Figure 15 we exhibit the best-performing three robots in all five lineages for the Lamarckian and Darwinian scenarios. We can see that in the Darwinian scenario, the morphologies are more elongated, while in the Lamarckian system X- and T-shaped robots seem to occur more frequently. This relates to the plots in Figure 14 where the size and proportion tend to increase in the Lamarckian setup, whilst the coverage decreases compared to the Darwinian setup.

### 6.4. Changes in CPPNs During Learning and Evolution

**Figure 16:**
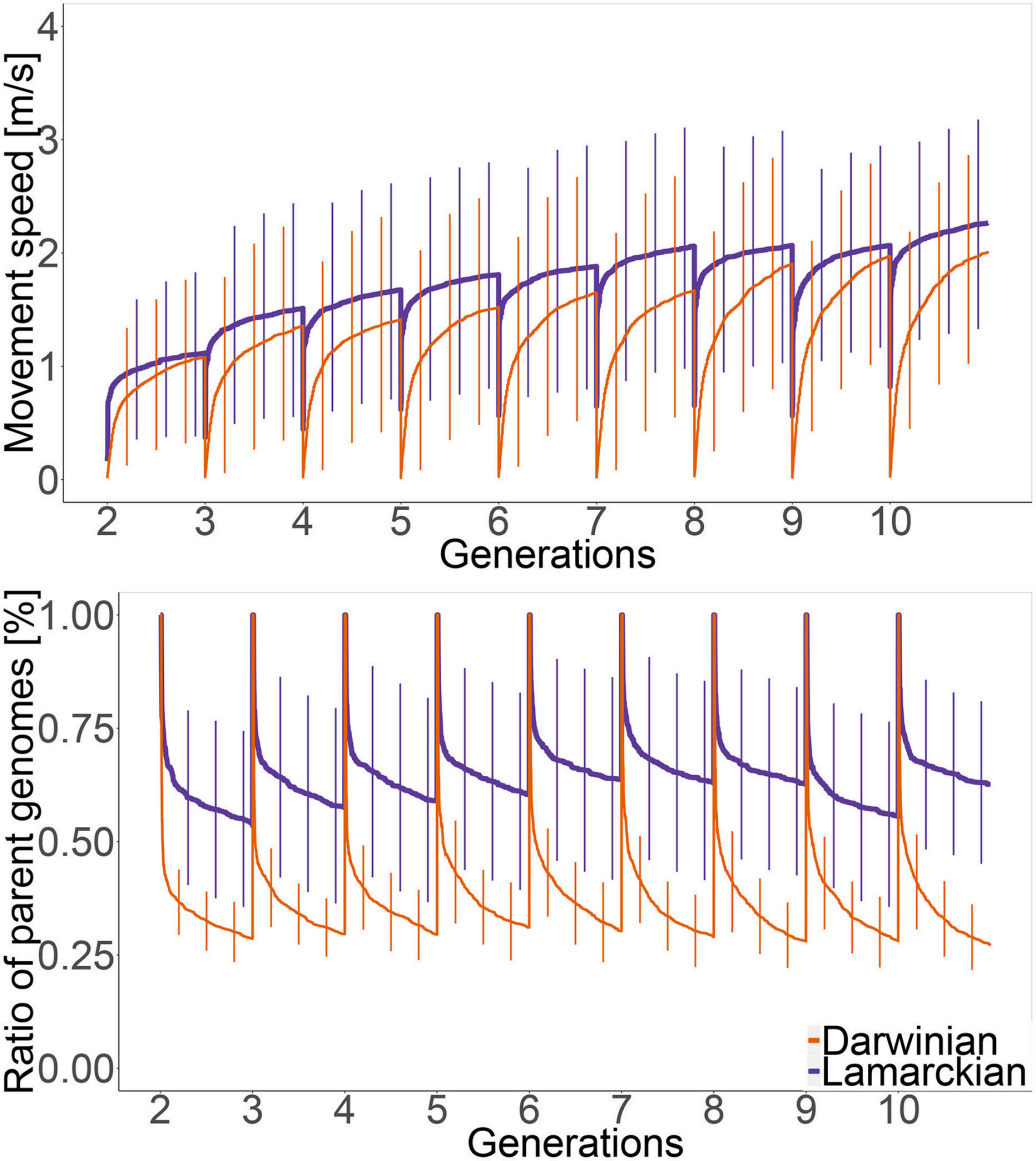
Development of speed (top plot) and the percentage of parental genetic material (bottom plot) within generations 2–10 in the Lamarckian (purple line) and the Darwinian (orange line) scenarios. The curves exhibit the averaged values of all robots in all lineages, the vertical bars are the standard deviations.

Finally, let us look beyond morphological issues and investigate how the rate of change in CPPN structure relates to performance. In Figure 16 the upper plot compares the performance trends, while the lower plot shows the ratio of parental genetic material for both the Darwinian and the Lamarckian setups. We can clearly see that in the Darwinian scenario parental genetic material is disappearing much faster than in the Lamarckian system. This can be explained by observing that the well-adapted inherited CPPNs of a “Lamarckian parent” need fewer modifications to adapt to a new body than the CPPNs of a “Darwinian parent.” The trend is also in line with the performance trend wherein every generation, in general, the Lamarckian setup outperformed the Darwinian.

## 7. Concluding Remarks

The long-term goal of our research is a technology to produce highly adapted robots for many possible environments and tasks. Our approach toward this goal is evolutionary, thus we are interested in robot systems where both morphologies and controllers undergo evolution. It is important to note that morphologically evolving robot systems need to include a learning period right after ‘birth' to acquire a controller that fits the newly created body. For efficiency reasons, the number of trials should be kept as low as possible. In this paper, we investigate the possibility of reducing the learning times by seeding the infant robot with the learned controllers of the parents. In particular, we address three research questions in the context of morphologically evolvable modular robots and locomotion as the main task: (1) Does the inheritability of learned features provide an advantage? (2) What does this advantage depend on? (3) Does Lamarckian evolution lead to different morphologies?

Our findings can be summarized in the following observations. First and foremost, it is possible to learn a proper gait with a relatively low number of trials (about 100 in our system) that make online learning in real time practicable. Secondly, we have confirmed the benefits of Lamarckian evolution. The main message of this paper is thus: Inheriting learned parental controllers speeds up infant learning, hence, using a Lamarckian set-up is advisable.

The added value of Lamarckism turned out to depend on the available learning budget, i.e., the allowable number of trials. If the learning budgets are limited (and in practice they always are), then starting off infant robot learning with inherited controllers makes a big difference.

Another interesting result is the correlation between the morphological similarity between the parents and the benefit of inheriting their controllers. Our data show that the benefits for the offspring are higher if the parents are more similar. This can play a significant role in future implementations since it can support educated guesses about how well the parental controllers will perform on the offspring robot. This issue deserves further research, for instance into autonomous mate selection mechanisms, where would-be parents do not rely on an abstract fitness value, but visually inspect each other. Such mechanisms that allow mate selection without a central authority using only locally observable information could be essential ingredients in real-world evolutionary robot systems of the future.

Last, but not least, we can draw additional conclusions considering the evolved morphologies. Remarkably, even though the difference between the Darwinian and Lamarckian versions only concern the controllers, the final impact is also visible in the space of morphologies.

Naturally, our results are based on one system only, and the advantages of inheriting parental controllers will depend on the given environment, the robot design, and the task. Nevertheless, the empirical evidence fully supports the common sense expectation that bootstrapping infant learning by inheriting learned parental controllers is a highly advisable option for the design of evolutionary robotic systems.

## Data Availability

The datasets generated and analyzed for this study is stored at the Vrije Universiteit Amsterdam repository ssh.data.vu.nl, and can be found in the 2018-frontiers-jelisavcic-lamarckian directory, available on request. The source code of the simulator used in this research is available on GitHub: https://github.com/ci-group/revolve/.

## Author Contributions

MJ conducted experiments, analyzed the data, wrote article structure. KG supervised experiments, analyzed the data, wrote article sections. EH supervised experiments, initially proposed part of the mechanism that was used, wrote article parts. AE supervised experiments, guided the group, wrote article parts, and formulated the conclusions.

### Conflict of Interest Statement

The authors declare that the research was conducted in the absence of any commercial or financial relationships that could be construed as a potential conflict of interest.

## Footnotes

^1^Note that Lamarckian evolution of morphologies is possible if robots have the ability of self-modification.

^2^http://robogen.org/

^3^http://robogen.org/docs/guidelines-for-writing-a-robot-text-file/

^4^In the standard specification, components are attached to a parent component only by the slot 0.

^5^The source code for Revolve can be found at https://www.github.com/ci-group/revolve

^6^In reality, a HyperNEAT run is terminated after 1,000 evaluations, but the performance is cross-sected after 10, 100 and 1,000 evaluations.

^7^Available online at https://youtu.be/GaxMlntR3IA
