# Supplementary material for: Lamarckian Evolution of Simulated Modular Robots
Source: Front Robot AI. 2019 Feb 18;6:9. doi: 10.3389/frobt.2019.00009 (PMC7805734; doi:10.3389/frobt.2019.00009)
Supplement: Supplementary file 2 [file Data_Sheet_1.pdf]

1 GENOME RECOMBINATION AND MUTATION

The purpose of this appendix is to illustrate the recombination and mutation operators we selected for the mating process. For clarity, we consider less complex morphologies than the ones typically evolved in the experiments.

When the recombination operator is applied to the parent genomes shown in Figure 17, sub-trees are randomly exchanged, resulting in an offspring displayed in Figure 18.

Finally, the mutation operator may make a random mutation on the offspring - preferably with substantially low probability. Note that as long as the node is of valid format (i.e any valid robot part in the definition of RoboGen) the mutation is valid as well, meaning that it does not need to follow any paradigm (such as colour) from the parent genomes.

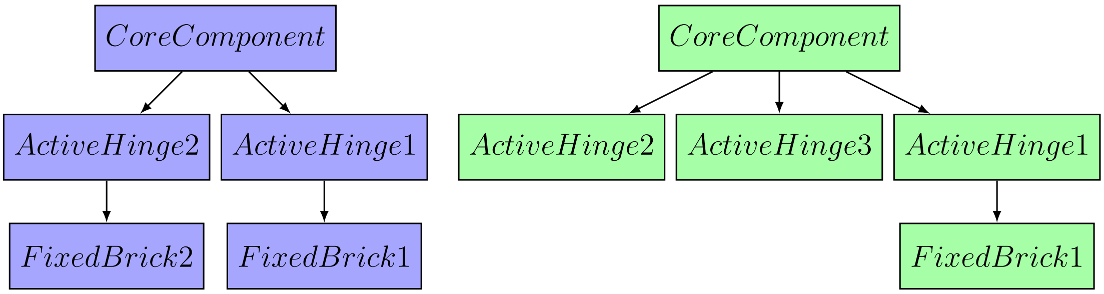

Figure S1. Tree representation of parent genomes.

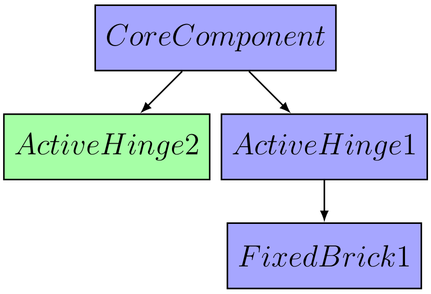

Figure S2. Offspring after recombination operator is applied.

2 MORPHOLOGICAL DESCRIPTORS

The Tree Edit Distance (TED) algorithm defined by Zhang and Shasha (1989)[8] quantifies the differences in tree structures for pairs of robot genomes. The algorithm applies the following cost rules:

- Adding, removing or modifying a node in a tree to a different type has a cost of 1;
- Moving a node to a different parent has a cost of 1.

The morphological descriptors used for quantifying robot morphologies are drawn from Miras et al. (2018). Here we only summarise definitions of the descriptors for clarity:

Branching. This descriptor captures how the attachments of the modules are grouped together in the body, and envisions to measure whether the components of the body are more spread or agglomerated. It is defined with Eq. (1):

$$B = \begin{cases} \frac{b}{b_{max}}, & \text{if } m >= 5 \\ 0 & \text{otherwise} \end{cases} \tag{1}$$

where m is the total number of modules in the body, b the number of modules that are attached on all four faces, and $b_{max} = \lfloor (m-2)/3 \rfloor$ – the maximum possible number of modules that can be attached on four faces in a body of m modules.

Number of Limbs. This describes the number of extremities of a body:

$$\begin{aligned} L &= \begin{cases} \frac{l}{l_{max}}, & \text{if } l_{max} > 0 \\ 0 & \text{otherwise} \end{cases} \\ l_{max} &= \begin{cases} 2 * \lfloor \frac{(m-6)}{3} \rfloor + (m-6) \pmod 3 + 4, & \text{if } m >= 6 \\ m-1 & \text{otherwise} \end{cases} \end{aligned} \tag{2}$$

where m is the total number of modules in the body, l the number of modules which have only one face attached to another module (except for the core-component) and l_{max} is the maximum amount of modules with one face attached that a body with m modules could have.

Length of Limbs. Describes how extensive the limbs of the body are and is defined with Eq. (3):

$$E = \begin{cases} \frac{e}{e_{max}}, & \text{if } m >= 3 \\ 0 & \text{otherwise} \end{cases} \tag{3}$$

where m is the total number of modules of the body, e is the number of modules which have two of its faces attached to other modules (except for the core-component), and $e_{max} = m - 2$ – the maximum amount of modules that a body with m modules could have with two of its faces attached to other modules, if containing the same amount of modules arranged in a different way[9]

Coverage. Describes how full is the rectangular envelope around the body. The greater this number, the less empty space there is between neighbour modules. It is defined as Eq. (4):

$$C = \frac{m}{m_{area}} \tag{4}$$

[8] A Python implementation of this algorithm can be found on `https://github.com/timtadh/zhang-shasha`.

[9] The types of modules would not have to be necessarily the same, as long as the body had the same amount of modules.

where m is the total number of modules of the body, and $m_{area} = m_l * m_w$ – the supported number of modules in the area of the body, with m_l being the number of modules that would fit in a column as long as the length of the body, and m_w the number of modules that would fit in a row as long as the width of the body.

Joints. This describes how movable the body is and is defined with Eq. (5):

$$J = \begin{cases} \frac{j}{j_{max}}, & \text{if } m >= 3 \\ 0 & \text{otherwise} \end{cases} \tag{5}$$

where m is the total number of modules of the body, j is the number of effective joints, i.e., joints which have both of its opposite faces attached to the core-component or a brick, and $j_{max} = \lfloor (m-1)/2 \rfloor$ – the maximum amount of modules with two opposite faces attached that a body with m modules could have, in an optimal arrangement.

Proportion. This describes the 2D ratio of the body and is defined with Eq.(6):

$$P = \frac{p_s}{p_l} \tag{6}$$

where p_s is the shortest side of the body, and p_l is the longest side, after measuring both dimensions of length and width of the body.

Symmetry. This describes the reflexive symmetry of the body with Eq.(7):

$$Z = \max_{z_v z_h} \tag{7}$$

where $z_h = o_h/q_h$ – is the horizontal symmetry, and $z_v = o_v/q_v$ – the vertical symmetry. For calculating each of these symmetry values, a referential centre for the body is defined as *the CoreComponent*. For both horizontal h and vertical v axes, a spine is determined as a line dividing the body into two parts according to the centre and this axis. Each value is the number o of modules that have a mirrored module on the other side of the spine (each match of modules accounts for two), divided by the total number q of compared modules. The spine is not accounted in the comparison.

Size. This describes the extent of the body in terms of the number of modules and is defined with Eq.(8):

$$S = \frac{m}{m_{max}} \tag{8}$$

where m is the total number of modules in the body and m_{max} the maximum number of modules permitted in each body.

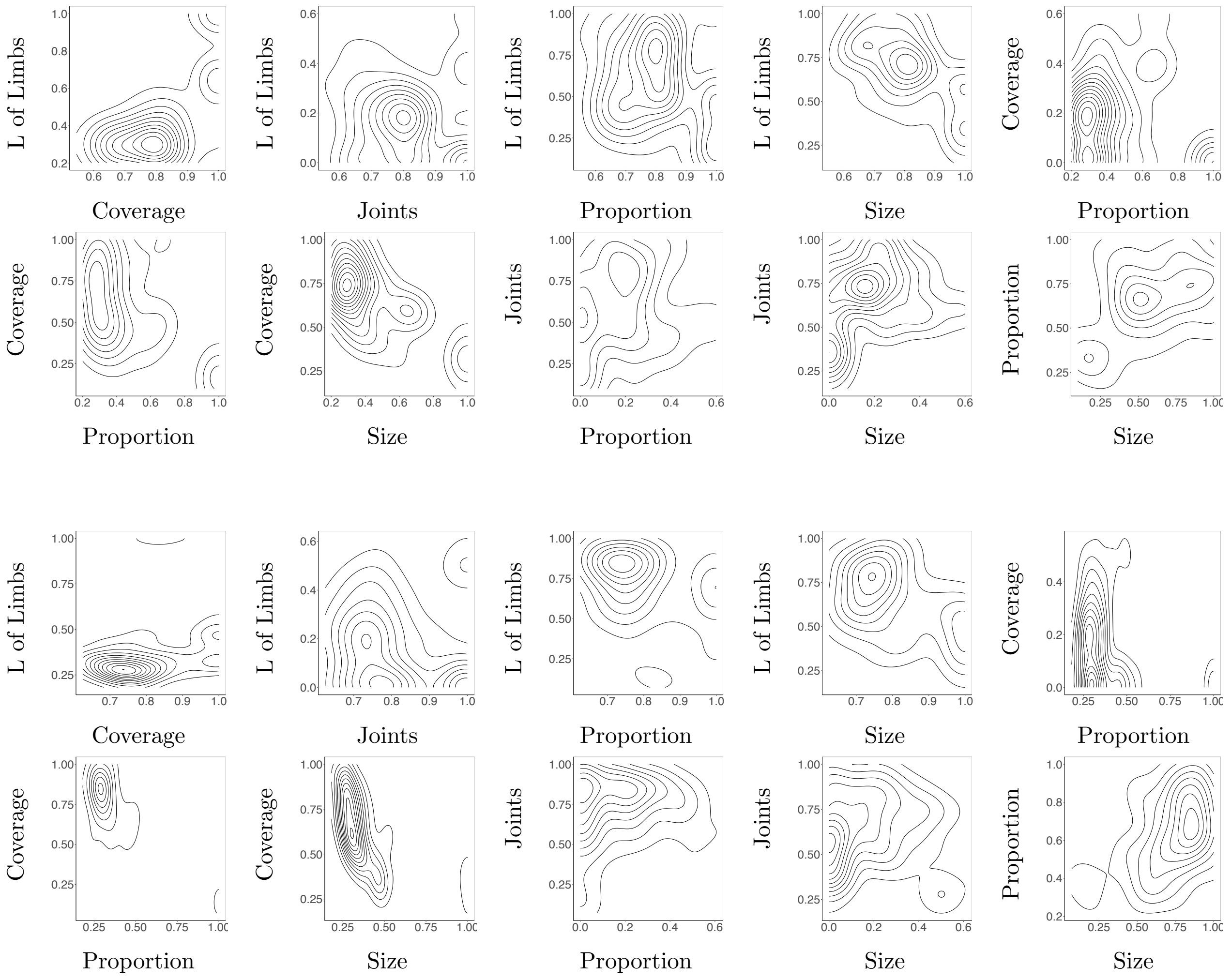

Figure S3. Density areas for the prominent morphological descriptors in the 10th generation of both Darwinian (top) and Lamarckian (bottom) regimes. Every plot represents the density correlation between two descriptors.

3 EXPERIMENTAL SETTINGS

Simulator		
Parameter	*Description*	*Value*
$engine$	Physics engine	ODE (quick solver)
t_{step}	Maximum step size of	0.003 seconds
$iter$	Iterations per timestep	1
$torque$	Torque of the motors	0.1 Nm
m_{block}	Weight of a *FixedBrick* module	32 g
m_{joint}	Weight of a *ActiveJoint* module	16 g
	**Morphological crossover**	
**Parameter**	**Description**	**Value**
$\|\mathcal{R}\|_{\text{max}}$	Maximum number of nodes	50
$\|\mathcal{R}\|_{\text{min}}$	Minimum number of nodes	5
o_{max}	Maximum number of outputs	50
μ_{parts}	Mean of randomly generated parts $\mathcal{N}(\mu_{\text{parts}}, \sigma^2_{parts})$	12
σ_{parts}	Standard deviation of randomly generated parts	5
$p_{\text{remove subtree}}$	Probability of removing subtree	0.05
$p_{\text{duplicate subtree}}$	Probability of duplicating subtree	0.1
$p_{\text{swap subtree}}$	Probability of swapping subtree	0.05
$p_{\text{remove hidden neuron}}$	Probability of removing hidden neuron	0.05
$p_{\text{remove neural connection}}$	Probability of removing neural connection	0.05
	**HyperNEAT**	
**Parameter**	**Description**	**Value**
P_{cppn}	CPPNs population size	10
O_{max}	Maximal number of offspring CPPNs	9
p_{weight}	Probability of weight mutation	0.8
σ_{weight}	Standard deviation of weight mutation	0.8
p_{mutate}	Probability of parameter mutation	0.8
σ_{param}	Standard deviation of parameter mutation	0.8
$p_{augment}$	Probability of structural augmentation	0.8
p_{remove}	Probability of structural removal	0
G_{max}	Number of evaluated CPPN generations	100
$p_{init.struct.mutation}$	Probability of structural augmentation applied on a starting network (0 for random weights/parameters)	0
$p_{interspecies}$	Probability of crossover with other-specie CPPNs	0.01
t_{eval}	Evaluation time	30 s
	**Experiments**	
**Parameter**	**Description**	**Value**
$Init$	Initialisation	random
P_{robots}	Robot population size	20
$Select$	Parent selection	2 parents with binary tournament
N_{gen}	Max. number of generations (i.e. termination criterion)	10

Table S1. Parameter values applied for the simulator, body crossover method and HyperNEAT learner used in the experimental setup.
